# Tumor volume of resectable gastric adenocarcinoma on multidetector computed tomography: association with N categories

**DOI:** 10.6061/clinics/2016(04)04

**Published:** 2016-04

**Authors:** Hang Li, Xiao-li Chen, Jun-ru Li, Zhen-lin Li, Tian-wu Chen, Hong Pu, Long-lin Yin, Guo-hui Xu, Zhen-wen Li, Jing Reng, Peng Zhou, Zhu-zhong Cheng, Ying Cao

**Affiliations:** I Sichuan Academy of Medical Sciences and Sichuan Provincial People's Hospital, Department of Radiology, Chengdu, Sichuan, China; II Sichuan Cancer Hospital and Institute & The Second People's Hospital of Sichuan Province, Department of Radiology, Chengdu, Sichuan, China; III West China Hospital of Sichuan University, Department of Out-patient, Chengdu, Sichuan, China; IV Affiliated Hospital of North Sichuan Medical College, Sichuan Key Laboratory of Medical Imaging and Department of Radiology, Nanchong, Sichuan, China

**Keywords:** Gastric adenocarcinoma, Lymph node metastasis, N categories, MDCT, Gross tumor volume

## Abstract

**OBJECTIVE:**

To determine whether the gross tumor volume of resectable gastric adenocarcinoma on multidetector computed tomography could predict the presence of regional lymph node metastasis and could determine N categories.

**MATERIALS AND METHODS:**

A total of 202 consecutive patients with gastric adenocarcinoma who had undergone gastrectomy 1 week after contrast-enhanced multidetector computed tomography were retrospectively identified. The gross tumor volume was evaluated on multidetector computed tomography images. Univariate and multivariate analyses were performed to determine whether the gross tumor volume could predict regional lymph node metastasis, and the Mann-Whitney U test was performed to compare the gross tumor volume among N categories. Additionally, a receiver operating characteristic analysis was performed to identify the accuracy of the gross tumor volume in differentiating N categories.

**RESULTS:**

The gross tumor volume could predict regional lymph node metastasis (*p*<0.0001) in the univariate analysis, and the multivariate analyses indicated that the gross tumor volume was an independent risk factor for regional lymph node metastasis (*p*=0.005, odds ratio=1.364). The Mann-Whitney U test showed that the gross tumor volume could distinguish N0 from the N1-N3 categories, N0-N1 from N2-N3, and N0-N2 from N3 (all *p*<0.0001). In the T1-T4a categories, the gross tumor volume could differentiate N0 from the N1-N3 categories (cutoff, 12.3 cm^3^), N0-N1 from N2-N3 (cutoff, 16.6 cm^3^), and N0-N2 from N3 (cutoff, 24.6 cm^3^). In the T4a category, the gross tumor volume could differentiate N0 from the N1-N3 categories (cutoff, 15.8 cm^3^), N0-N1 from N2-N3 (cutoff, 17.8 cm^3^), and N0-N2 from N3 (cutoff, 24 cm^3^).

**CONCLUSION:**

The gross tumor volume of resectable gastric adenocarcinoma on multidetector computed tomography could predict regional lymph node metastasis and N categories.

## INTRODUCTION

Gastric cancer is one of the most common cancers and the second most common cause of cancer-related mortality worldwide, and most gastric cancers are gastric adenocarcinoma 1,2. In the United States, 22,220 new cases of this malignancy were estimated to have occurred in 2011, resulting in 10,990 expected deaths 3. Lymph node metastasis (LNM) is a well-established critical prognostic factor, and accurate staging of LNM is desirable for preoperative treatment 4,5. According to the 7th edition of the American Joint Committee on Cancer (AJCC) staging system, released in 2010, at least 16 regional lymph nodes should be assessed pathologically to determine the N category 6. Although an increasing incidence of LNM has been observed in patients who undergo extended lymphadenectomy, postoperative morbidity and mortality remain high 7,8-9. Therefore, extended lymphadenectomy cannot be recommended for the treatment of all patients with gastric cancer, and accurate noninvasive assessment of LNM and the N category plays an important role in determining whether these patients should undergo complete resection of the primary tumor and more extensive lymphadenectomies.

Currently, the tools for preoperative assessment of LNM and the N category of gastric cancer include endoscopic ultrasonography (EUS), computed tomography (CT) and positron emission tomography/computed tomography (PET/CT). EUS can distinguish the different layers that compose the gastric wall and can be used to visualize the perigastric lymph nodes via a miniaturized ultrasonography (US) probe. However, this approach is invasive and lacks the capacity for panoramic investigation, and a sufficiently stenotic stomach may prevent passage of the endoscope. A previous study reported that the diagnostic performance of EUS for LNM identification is relatively unreliable, and the overall accuracy of EUS in determining the N category was only 66% in another study 10,11. In the latter study, on CT, lymph nodes were considered positive for metastasis if they were ≥8 mm along the short-axis diameter 11. Because CT could not reliably detect small LNM, the diagnostic accuracy was approximately 62.8% 11. In addition, PET/CT can help to assess regional LNM, but with a low diagnostic accuracy, or 58%, because PET/CT cannot detect regional LNM in patients with early gastric cancer 12. Previous studies have shown that the gross tumor volume (GTV) of esophagogastric junction adenocarcinoma and esophageal cancer measured on multidetector computed tomography (MDCT) is associated with regional LNM and N categories 13,14. Hallinan et al also reported that the GTV was moderately accurate in predicting the N stage 15. To our knowledge, however, there have been no reports regarding the utility of the GTV of resectable gastric adenocarcinoma measured on MDCT in predicting regional LNM and N categories. Thus, we aimed to retrospectively assess whether the GTV of resectable gastric adenocarcinoma measured on MDCT could predict regional LNM and N categories.

## MATERIALS AND METHODS

This study was approved by the institutional ethics committee, and written informed consent was obtained from each patient.

A total of 250 consecutive patients with gastric adenocarcinoma diagnosed at our institution were retrospectively recruited into the study between June 2013 and July 2014. Of the 250 participants, 17 patients who had not undergone surgery, including 3 patients with other severe disease, 8 patients with distant metastasis, and 6 patients with direct invasion of an adjacent organ; 26 patients who had undergone radiation therapy and preoperative neoadjuvant chemotherapy; and 5 patients who had images of poor quality, were excluded from this study. Consequently, this study involved 202 patients.

In total, 141 of the 202 patients (69.8%) were males (median age, 61 years; age range, 22-79 years), and 61 (30.2%) were females (median age, 60 years; age range, 33-78 years). All patients underwent endoscopic biopsy and preoperative contrast-enhanced CT. Subsequently, the included patients underwent standard operative procedures: namely, distal subtotal or total gastrectomy with D2 or more extended lymphadenectomy. The interval between CT and surgery was less than one week. Among the 202 patients, a total of 3756 lymph nodes were removed, with a mean of 18 nodes (range, q7-30) for each patient. According to the postoperative pathologic examination, 136 patients had LNM, whereas 66 patients did not. According to the postoperative histopathology and AJCC criteria 6, the tumors were located in the upper one-third of the stomach in 55 patients, the middle one-third in 50 patients and the lower one-third in 97 patients. Tumor histology was classified into two groups: the differentiated group (well- or moderately differentiated adenocarcinoma) in 129 patients and the undifferentiated group (poorly differentiated adenocarcinoma) in 73 patients. Primary tumors were classified as being in the T1 category in 19 patients, the T2 category in 40, the T3 category in 15, and the T4a category in 128. The dissected lymph nodes were classified into the N0 category in 66 patients, the N1 category in 39, the N2 category in 43, and the N3 category in 54. The presence of vascular or lymphatic invasion was observed in 81 patients.

### Contrast-enhanced MDCT

All patients were scheduled to undergo enhanced 64-section MDCT (Light-Speed VCT; General Electric Health Care, Chalfont St. Giles, United Kingdom). Before CT image acquisition, the patients ingested effervescent granules along with 500 mL of water to distend the stomach with gas. The patients were then examined by CT, and the CT data acquisition was performed in the arterial phase (25-30 s) and the portal-venous phase (60-70 s), covering the entire stomach in the arterial phase and the entire abdomen and pelvis after injection of contrast material (Ultravist 300, Iopamidol; Bayer Healthcare, Berlin, Germany). The CT scanning variables were 120 kVp, 200-380 mA, a section thickness of 2 mm, and a reconstruction interval of 2 mm. Scanning was performed during the arterial and portal venous phases, and the anatomic coverage was from the apex of the lungs to the pelvic cavity. The data were directly interfaced and forwarded to the General Electric Advantage Workstation 4.4 (Advantage Workstation version 4.4; General Electric Healthcare).

### GTV measurement

**Figure 1:**
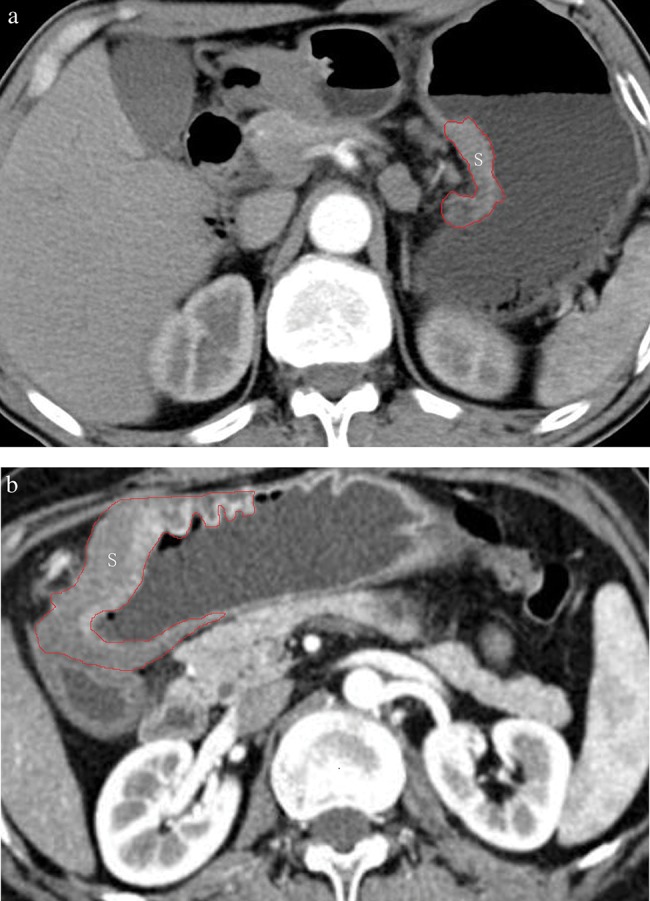
Transverse contrast-enhanced multidetector computed tomography (CT) images in a 55-year-old man (a) and a 65-year-old man (b) with gastric adenocarcinoma. The tumor area (S) is depicted on the axial contrast-enhanced CT image.

The GTV was measured at a window width of 380 HU and a window level of 50 HU. In particular, the GTV was obtained by multiplying the sum of all tumor areas by the section thickness according to a protocol in a previous report 13,16,17. For delineation of the tumor area, a gastric wall thickness ≥5 mm on transverse imaging with the stomach distended was regarded as abnormal 18. The tumor area was depicted on each axial enhanced CT image (Figure 1) and was automatically calculated by the software. After assessing the whole tumor, each contiguous transverse tumor area was summed to obtain the GTV. The time needed to obtain the GTV was approximately 200 s on average (range, 100-350 s).

To maintain the accuracy of the measurements, 2 experienced radiologists working in consensus were trained in measuring the GTV randomly in another 20 patients by a radiologic professor. All tumor measurements were repeated one month later to test the interobserver reproducibility of the measurement of the GTV.

### Statistical analysis

All statistical analyses were carried out with SPSS (version 17.0, SPSS, Chicago, IL, United States). *P*<0.05 was considered to represent a significant difference.

The CT data of the 202 patients with gastric adenocarcinoma were used to test the interobserver reproducibility of the measurements. In these 202 patients, the precision of the replicated GTV measurements was assessed using the coefficient of variation (CV) (standard deviation / mean × 100). When the %CV was less than 10%, interobserver variability was considered to be small, and the averaged value of the two observers' measurements was regarded as the final GTV. If the %CV exceeded 10%, another two measurements were performed by the previous observers, and the average of the four measurements was used as the final GTV.

Univariate associations between LNM and both the GTV and clinicopathological factors were analyzed using the chi-square test (or Fisher's exact test when appropriate). Multivariate logistic regression analyses were used to assess the associated risk factors for LNM. GTV values were compared between patients stratified by N category using non-parametric Mann-Whitney U tests together with Bonferroni correction for multiple comparisons. A receiver-operating characteristic (ROC) analysis was also performed to determine the threshold GTV values for differentiation of N categories.

## RESULTS

### Interobserver variability of measuring tumor volume

For the first evaluation in this cohort, the mean GTV was 32.25±29.25 cm^3^ (range, 2.3-189.3 cm^3^). For the repetitive measurement, the mean GTV was 30.89±27.43 cm^3^ (range, 2.5-191.6 cm^3^). As for the precision of the CT measurements of the GTV, the CV was 5% (range, 1-14.6%). Therefore, the CV was less than 10%, and the interobserver variability of the GTV measurement was small, so the average value of the two measurements was regarded as the final GTV. However, for the two measurements in four patients, the CV exceeded 10; therefore, two additional measurements were obtained, and the average of the four measurements was used as the final GTV.

### Univariate and multivariate analyses of correlation of both clinicopathological factors and GTV with LNM

Based on the possible clinicopathological factors for predicting LNM, including age, gender, anatomical distribution, histologic type, T category, GTV and lymphatic or vascular invasion, the details of the univariate analysis are illustrated in Table 1. Based on the univariate analysis, histologic type, T category, GTV and lymphatic or vascular invasion showed an association with LNM. In particular, LNM was found more frequently in the patients with an undifferentiated histologic type than in those with a differentiated histologic type (*p*=0.004), in patients with a deeper tumor depth than in those with a lesser tumor depth (*p*<0.0001), in patient with a GTV ≥14.5 cm^3^ than in those with a GTV <14.5 cm^3^ (*p*<0.0001), and in patients with lymphatic or vascular invasion than in those without this invasion (*p*<0.0001). However, there were no significant associations between LNM and age, gender, or tumor anatomical distribution (*p*=0.506, 0.185, and 0.062, respectively).

Regarding the multivariate analysis, T category, GTV and lymphatic or vascular invasion were found to be independent risk factors for LNM. The GTV (*p*=0.005, odds ratio (OR)=1.364, and 95% confidence interval (CI) for OR of 1.015-2.438), T stage (*p*<0.0001, OR=2.337, and 95% CI of 1.519-3.596) and lymphatic or vascular invasion (*p*<0.0001, OR=9.886, and 95% CI of 2.505-39.24) of the primary tumor were associated with regional LNM.

### Correlation between N categories and GTV

Table 2 summarizes the correlation between the N categories and the GTV. The GTV could help to distinguish the N1 from the N2 category (*p*=0.004), N1 from N3 (*p*<0.0001), N2 from N3 (*p*<0.0001), N0 from N1-N3 (*p*<0.0001), N0-N1 from N2-N3 (*p*<0.0001), and N0-N2 from N3 (*p*<0.0001).

In addition, most of the patients in our study were in the T4a category. Therefore, we focused on investigating the GTV in the patients in the T4a category according to N categories (Table 2). In total, 114 of the 128 patients (89.1%) had LNM, and 14 of the 128 patients (10.9%) did not have LNM. In all, 26 of the 114 patients were classified into the N1 category, 39 patients were classified into the N2 category, and 49 patients were classified into the N3 category. In the T4a category, the GTV could help to distinguish between N1 and N2 (*p*=0.015), N1 and N3 (*p*<0.0001), N2 and N3 (*p*<0.0001), N0 and N1-N3 (*p*<0.0001), N0-N1 and N2-N3 (*p*<0.0001), and N0-N2 and N3 (*p*<0.0001).

### ROC analyses of accuracy of GTV of gastric adenocarcinoma in differentiating N categories

**Figure 2:**
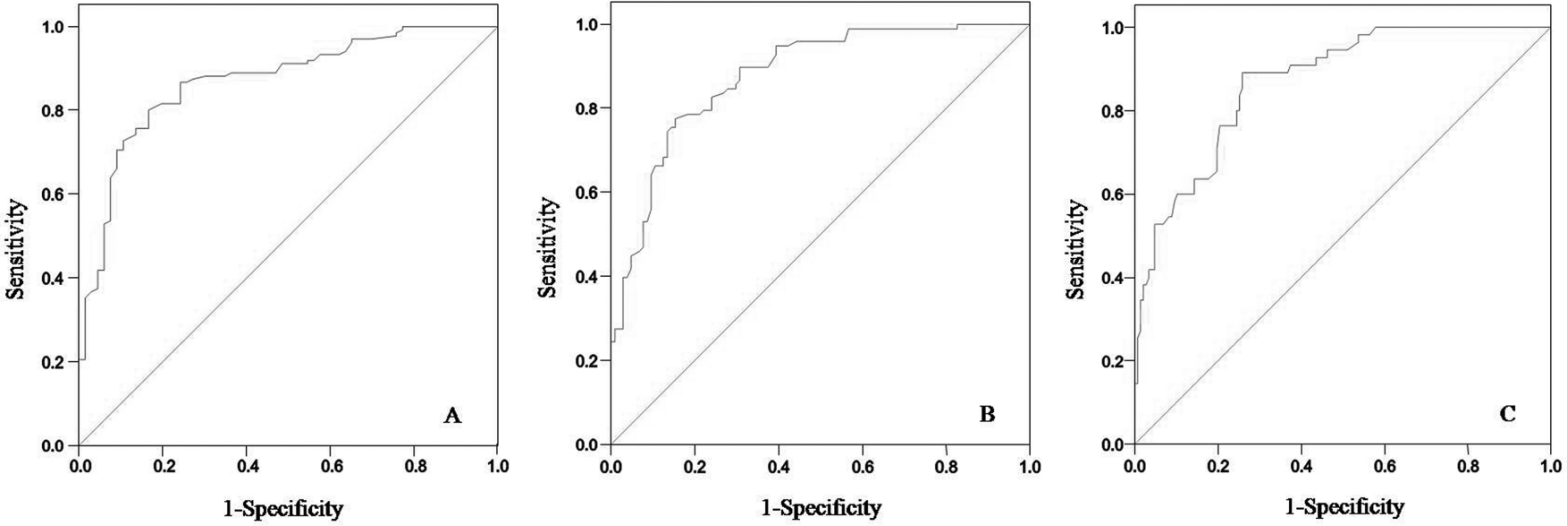
Receiver operating characteristic (ROC) curves of the accuracy of the gross tumor volume (GTV) in differentiating N categories in patients with resectable gastric adenocarcinoma in the T1-T4a categories. The ROC curve shows that the GTV could help to differentiate N0 from N1-N3 (A), N0-N1 from N2-N3 (B), and N0-N2 from N3 (C) by using GTV cutoff values of 12.30 cm^3^, 16.65 cm^3^ and 24.60 cm^3^, respectively.

Using ROC analysis of the T1-T4a categories, we found that the GTV could help to differentiate N0 from the N1-N3 categories (cutoff, 12.3 cm^3^), N0-N1 from the N2-N3 (cutoff, 16.6 cm^3^), and N0-N2 from N3 (cutoff, 24.6 cm^3^) (Figure 2). In the T4a category, the GTV could help to differentiate N0 from the N1-N3 categories (cutoff, 15.8 cm^3^), N0-N1 from N2-N3 (cutoff, 17.8 cm^3^), and N0-N2 from N3 (cutoff, 24 cm^3^) (Figure 3). The diagnostic efficiency, as assessed based on the area under the ROC curve, sensitivity, specificity, the positive predictive value, the negative predictive value and the accuracy of the GTV in differentiating the N categories, is shown in Table 3.

## DISCUSSION

LNM is known to be one of the major negative prognostic factors for patients with resectable gastric cancer after curative surgery 19. Radical gastrectomy with removal of regional lymph nodes has been considered as the standard for the treatment of curable gastric cancer 20. However, for many years, it has been debated whether extended lymph node dissection is beneficial for gastric cancer. Recently, D2 lymphadenectomy has become the standard treatment for curable gastric cancer in eastern Asia 21. However, previous research has shown that patients with advanced gastric cancer benefit from more extensive lymph node dissection 22. Moreover, extended lymphadenectomy may detect small LNM that is difficult to diagnose preoperatively. Theoretically, removal of a wider range of lymph nodes improves staging accuracy and increases the chances for cure, but its contribution to prolonged survival remains unclear. More extensive surgery can also contribute to more operation-related complications and mortality. Moreover, previous studies reported that neoadjuvant chemotherapy could help to decrease the N categories before surgical resection 23,24. Therefore, it is essential to accurately preoperatively diagnose LNM and the N category to determine whether patients with gastric cancer should receive gastrectomy alone or combined with D2 lymphadenectomy or more extensive lymphadenectomies and/or neoadjuvant chemotherapy before surgery. Most variables related to lymph node evaluation are pathological because it is very difficult to establish the N category preoperatively. At present, EUS, MDCT and PET/CT for diagnosis of the presence of LNM and determination of the N category are not the most reliable imaging modalities. In the current study, we retrospectively assessed the GTV of gastric cancer measured on MDCT and found that the GTV can be utilized to predict the presence of LNM and to determine the N category with a better diagnostic accuracy.

Previous studies have been performed to evaluate the risk of LNM in gastric cancer. Several prognostic factors, including depth of invasion, vascular invasion or lymphatic permeation, and tumor histologic type, have been demonstrated to be related to LNM in gastric cancer 19,25. Our study was consistent with these published reports. Certain studies have also reported that the GTV of esophageal squamous cell carcinoma and esophagogastric junction adenocarcinoma measured by CT or PET/CT is associated with regional LNM 13,14,26. Therefore, we wondered if the GTV of gastric cancer on CT could be helpful predicting regional LNM. In the present study, we found that the GTV remained an independent factor for predicting LNM when the T stage and the GTV were included in a multivariate model. Moreover, another study reported that the GTV was an important prognostic factor in patients who underwent curative resection for gastric cancer 27. It is also well known that LNM is one of the major negative prognostic factors for patients with resectable gastric cancer after curative surgery 19. Taking these findings into consideration, we presume that one of the key reasons for the GTV being an important prognostic factor in patients is its influence on regional LNM. This hypothesis should be confirmed in a future study.

The prediction of regional LNM in gastric cancer by EUS, CT and PET/CT has been studied. Mocellin et al reported that EUS had high diagnostic efficiency in staging the T category but that it was less reliable in predicting LNM, with a sensitivity of 0.69 and a specificity of 0.84 10. Hwang et al reported that detection of LNM by EUS was effective in 70.4% of cases (sensitivity: 19.3%, specificity: 96.3%) 11. Recently, MDCT scanners have begun to be widely used worldwide, allowing more detailed imaging with thinner section collimation. Certain previous investigators relied on different lymph node morphologic criteria for predicting LNM, although with accuracy remaining approximately 70% 11,28. As for PET/CT, Kim et al reported that the specificity was 100% for predicting LNM, although the sensitivity was low (41%) and the accuracy was 51% 29. In the present study, we found that the GTV measured on MDCT could help to predict LNM with a sensitivity of 81.6%, a specificity of 83.3% and an accuracy of 81.2%.

In the past, the patients with gastric category in the T4 category did not receive surgery 30. Recently, however, these patients have begun to receive surgery. According to previous reports, preoperative prediction of the presence of LNM and the number of lymph node metastases for T4 tumors is of particular importance in determining tumor resectability and the optimal extent of surgery 31,32. Because of the significant difference in the GTV of gastric cancer between N categories, we assessed whether the GTV can differentiate N categories within the T1-T4a categories. There were 128 patients in the T4a category in our study, so we also specifically used the GTV to differentiate N categories only in T4a category. For the T1-T4a categories, we found that the GTV could help to differentiate N0 from the N1-N3 categories (cutoff, 12.3 cm^3^), N0-N1 from N2-N3 (cutoff, 16.6 cm^3^), and N0-N2 from N3 (cutoff, 24.6 cm^3^). Additionally, in the present study, the accuracies of the GTV were higher than in a previous report 15 in terms of differentiating N0 from the N1-N3 categories (81.2% *vs* 75%), N0-N1 from N2-N3 (78.7% *vs* 74%), and N0-N2 from N3 (76.2% *vs* 75%). This difference may be due to the facts that the sample size in the current study was larger than that in the previous study and that there were many patients in the T4a category. In the T4a category in particular, the GTV could help to differentiate N0 from the N1-N3 categories (cutoff, 15.8 cm^3^), N0-N1 from N2-N3 (cutoff, 17.8 cm^3^), and N0-N2 from N3 (cutoff, 24 cm^3^). The potential mechanism for the effect of the GTV on N categories could be that the larger the GTV is; the deeper the tumor invasion of several layers of the stomach is; the more likely this invasion is to involve lymphatics in the submucosal layer; and therefore, the more frequent the incidence of LNM is.

There were several limitations in this study. First, this was a retrospective study. Additionally, we did not apply the thresholds identified in the current study to a completely new patient population and compare the preoperative N categories to the postsurgical staging and then calculate the accuracy of our recommended CT methodology. Second, it was occasionally difficult to distinguish tumors from neighboring organs, especially for tumors in the T4a category. To minimize this effect, each patient ingested effervescent granules within 10 mL of water to distend the stomach with gas before CT. Third, the 202 patients included in this study had an indication for surgery, whereas patients who had a contraindication for surgery were excluded from this study. However, our findings are applicable to resectable gastric adenocarcinoma. Finally, there was large variation of the GTV CI in the categories, and the OR of the GTV was only 1.364 in the multivariate analysis. The probable reason for these features is our relatively small sample size. Therefore, a larger sample size should be examined to validate our research in the future. Despite these limitations, all of the enrolled cases were staged based on postoperative histopathology, and our study demonstrated the potential use of MDCT as a tool to measure the GTV to predict LNM and to determine N categories. We will focus on our recommended thresholds for the GTV to predict the presence of LNM and to determine N categories in a second, prospective study.

In conclusion, we found that the GTV of resectable gastric adenocarcinoma measured on MDCT was associated with the presence of regional LNM and with N categories. In the T1-T4a categories, a sensitivity, a specificity and an accuracy of more than 76% were calculated by using GTV thresholds of 12.30 cm^3^, 16.65 cm^3^, and 24.60 cm^3^ for differentiating N0 from N1-N3, N0-N1 from N2-N3, and N0-N2 from N3, respectively. In the T4a category, GTV thresholds of 15.79 cm^3^, 17.75 cm^3^, and 24.0 cm^3^ could help to differentiate N0 from the N1-N3 categories, N0-N1 from N2-N3, and N0-N2 from N3, respectively. We believe that the GTV could help clinicians to quantitatively predict the presence of LNM and to determine N categories when choosing the optimal treatment modalities for individual cases.

## AUTHOR CONTRIBUTIONS

Hang L, Chen XL, Li JR, Li ZL, Chen TW, Pu H and Yin LL contributed to the conception and design of the study, to the generation, collection, assembly, analysis and interpretation of the data, to the drafting and revision of the manuscript and approved the final version of the manuscript. Xu GH, Li ZW, Reng J, Zhou P, Cheng ZZ and Cao Y contributed to the generation, collection, assembly, analysis and interpretation of the data and approved the final version of the manuscript.

## Figures and Tables

**Table 1:** . Univariate analysis of clinicopathological factors and gross tumor volume correlated with regional lymph node metastasis.

Variable	Lymph node metastasis		*p* value
		Negative (n=66)	Positive (n=136)	
Age*	58.63±10.13	59.58±10.89	0.506
Gender			0.185
Male	43(65.1)	98(72.1)	
Female	23(34.9)	38(27.9)	
Anatomical distribution			0.062
Upper 1/3	18(27.3)	37	
Middle 1/3	38(57.6)	12	
Lower 1/3	10(15.1)	87	
Histologic type			0.004
Differentiated	33(50)	96(70.6)	
Undifferentiated	33(50)	40(29.4)	
T category			<0.0001
T1	18(27.3)	1(0.7)	
T2	28(42.4)	12(8.8)	
T3	6(9)	9(6.6)	
T4a	14(21.3)	114(83.9)	
Gross tumor volume (cm^3^)			<0.0001
<14.5	55(83.3)	32(23.5)	
≥14.5	11(16.7)	104(76.5)	
Lymphatic or vascular invasion			<0.0001
Absent	63(95.4)	58(42.6)	
Present	3(4.6)	78(57.4)	

Note: The numbers in the parentheses are percentages.

*The data are the median ± standard deviation.

**Table 2:** Gross tumor volume of resectable gastric adenocarcinoma in patients stratified by N category.

N category	T1-T4a categories (n=202)	T4a category (n=128)
N0	5.40(2.98, 9.45)	11.47(3.56, 19.37)
N1	15.47(4.48, 20.87)	16.50(6.77, 24.75)
N2	26(15.20, 33.60)	26.25(15.75, 39.60)
N3	48.75(27, 110)	50.47(27, 112.77)
N0-N1	6.90(3.27, 15.75)	15.20(4.50, 23.50)
N0-N2	11.01(4.40, 23.50)	16.87(11.73, 32.12)
N1-N3	26.60(15.2, 48.56)	30(16, 52.9)
N2-N3	32.25(19.68, 61.20)	33(20.44, 72)

Note: The data are presented as the median (25th percentile, 75th percentile).

**Table 3:** Receiver operating characteristic analysis of accuracy of gross tumor volume of resectable gastric adenocarcinoma in detecting N categories.

Gross tumor volume cutoff	N category comparisons	AUC	Sensitivity (%)	Specificity (%)	PPV (%)	NPV (%)	Accuracy (%)
T1-T4a categories (n=202)							
12.30(cm^3^)	N0 vs N1-N3	0.870	81.6	83.3	90.8	67	81.2
16.65(cm^3^)	N0-N1 vs N2-N3	0.878	80	77.9	77.2	80.2	78.7
24.60(cm^3^)	N0-N2 vs N3	0.869	76.4	76.5	60	89.3	76.2
T4a category (n=128)							
15.79(cm^3^)	N0 vs N1-N3	0.790	77.2	78.6	96.7	30	77.3
17.75(cm^3^)	N0-N1 vs N2-N3	0.800	79.8	72.8	86.5	60.9	77.3
24.00(cm^3^)	N0-N2 vs N3	0.807	80	67	56.3	82.4	67.9

Note: AUC = area under the receiver operating characteristic curve, PPV = positive predictive value, NPV = negative predictive value.
